# Global establishment threat from a major forest pest via international shipping: *Lymantria dispar*

**DOI:** 10.1038/s41598-018-31871-y

**Published:** 2018-09-13

**Authors:** D. R. Paini, P. Mwebaze, P. M. Kuhnert, D. J. Kriticos

**Affiliations:** 1 CSIRO, Black Mountain, Clunies Ross St, Canberra, Australia; 2 CSIRO, Ecosciences Precinct, Dutton Park, Brisbane, Australia; 3 CSIRO Data61, Black Mountain, Clunies Ross St, Canberra, Australia

## Abstract

The global shipping network is widely recognised as a pathway for vectoring invasive species. One species of particular concern is *Lymantria dispar* (gypsy moth). Two subspecies, *L*. *d*. *asiatica* and *L*. *d*. *japonica* (herein referred to as Asian Gypsy Moth - AGM) are of considerable concern as ships arriving to a number of countries have been found carrying AGM egg masses. However, ships carrying AGM eggs can only threaten a country at ports located in a climatically suitable region. We present a CLIMEX model of climate suitability and combine this with international shipping to estimate the global threat from AGM. We find that for the USA more than half of international ships (approximately 18,000 ships) arrive to climatically suitable ports. Other countries with a large number of ships arriving to ports with suitable climates include Canada and Brazil. This is the first global analysis of the invasion threat from AGM, and we recommend countries focus AGM-inspection programs towards ships arriving at ports found within climatically suitable regions.

## Introduction

The global spread of invasive species presents significant economic risks to many countries^1,2^. The gypsy moth (*Lymantria dispar* L.) is a notorious invader, currently spreading through North America, severely damaging many forest tree species and is seen as a global threat to both commercial and native forest systems^3^. In the US, the economic impacts of one subspecies, the European gypsy moth (*L*. *dispar dispar* L.) are estimated to be in excess of $250 million per year^4^, and this is likely to increase as this species continues to spread through North America. Two other subspecies, the Asian gypsy moth (*L*. *dispar asiatica* Vinkovskij), found in China, the Korean peninsula and far East Russia, and the Japanese gypsy moth (*L*. *dispar japonica* Motschulsky), found in Japan, have not, as yet established outside their native range, but are of significant global concern. As such, a number of countries such as Australia^5^, New Zealand^6^, Canada^7^, and the US^8^ have established active phytosanitary protocols to prevent their establishment.

The females of these two subspecies, *L*. *d*. *asiatica* and *L*. *d*. *japonica*; (for brevity we hereafter refer to these two subspecies as Asian Gypsy Moth - AGM) are able to fly^9^, and are attracted to lights^10^, so are often attracted to sea ports at night. Further, females are indiscriminate in their choice of oviposition site and will often oviposit on ships docking in these ports^11^. These ships can therefore potentially transport gypsy moth eggs to other locations around the world, and a number of ships arriving into Australia, New Zealand, Canada, and the US from ports within the current gypsy moth distribution have been found with gypsy moth eggs attached to them^12^. Biosecurity authorities are concerned that these eggs could hatch on arrival, and a population could establish as there have already been a number of incursions in North America^13^ that were subsequently eradicated. A risk assessment framework has been developed that combines likelihood of a ship carrying AGM eggs, the likelihood of those eggs completing diapause in transit and hatching at a destination port, and the likelihood of AGM subsequently establishing^12,14,15^. While this work represents a more thorough risk assessment than provided in this paper, it is dependent on obtaining a ship’s log of ports visited, which is often not available to a country’s biosecurity authorities, or at least, are not routinely incorporated into operationalised inspection activities at ports.

For a country’s biosecurity agency, having an understanding of which ports are found within the potential distribution of these two subspecies can support targeted allocation of inspection resources. If a port is located in a region that does not have suitable climatic conditions for these two subspecies, there may be little value in inspecting ships for AGM egg masses as, even if they hatch on arrival, no populations will be able to establish. Previous researchers have modelled the potential distribution of this species^3,16^ in order to identify the countries or regions at risk of AGM establishment. Matsuki and colleagues generated a CLIMEX model of non-diapausing AGM populations and presented a risk map for Australia^16^. This model was fitted using a coarse representation of the known Eurasian distribution of AGM^17^. While non-diapausing eggs are a possibility, and has been shown in laboratory reared populations^18^, non-diapausing populations have never been reported in the field^16^. For border biosecurity staff then, this does not provide an appropriate risk profile, as eggs arriving on a ship would most likely be from diapausing populations and for those eggs to hatch and establish, suitable climatic conditions would need to be present for subsequent populations to complete diapause. Peterson and colleagues present a GARP model of the potential distribution of *L*. *dispar*, which indicates that these subspecies could potentially establish in not just moist temperate zones, but also in large tropical zones of South America, eastern Africa, and South East Asia^3^. Considering that the current distribution of these two subspecies does not include the subtropics, it seems unlikely that this model is accurately simulating the range-limiting factors for AGM.

With a view to providing port invasion risk information for a country’s biosecurity agency at the global scale, we refitted Matsuki’s CLIMEX model using the distribution points from^3^, to estimate those areas that are climatically suitable for AGM establishment. We then use the refined model to identify those countries at risk from AGM arrival and establishment via shipping pathways. Within these vulnerable countries, we then identified the specific ports located within climatically suitable coastal regions as well as the number and proportion of total international ships arriving to these vulnerable ports, in order to provide a first step in identifying those countries at greatest risk of invasion from AGM.

## Results

### CLIMEX model

**Figure 1:**
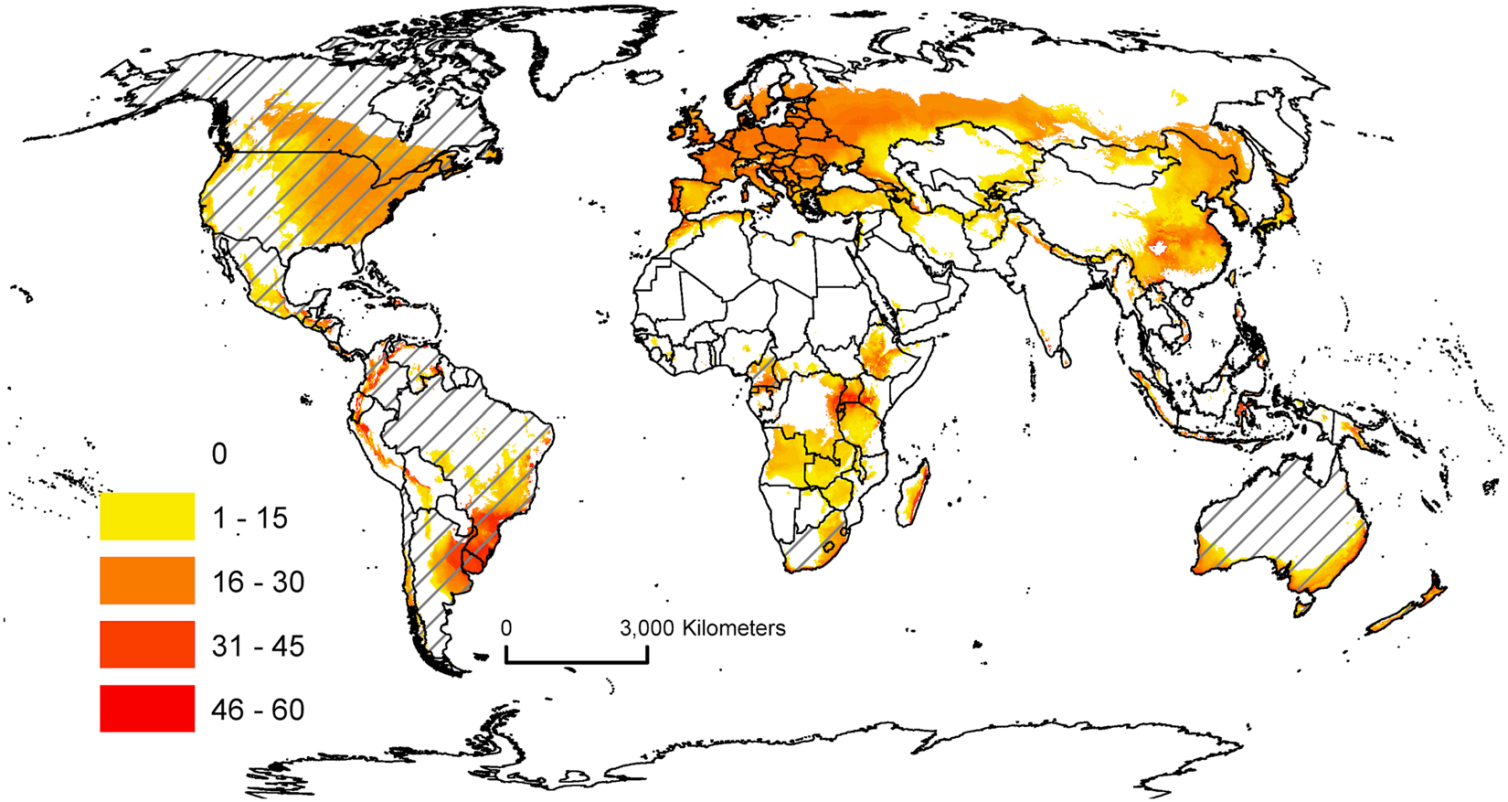
Global climate suitability for gypsy moth (*L*. *d*. *asiatica* & *L*. *d*. *japonica*) modelled using CLIMEX Ecoclimatic Index (EI). Cross-hatched countries are those with vulnerable ports (refer Table 1).

The modelled potential distribution of AGM spans the Mediterranean, warm Temperate and sub-tropical climate zones (Fig. 1). The potential distribution of AGM includes a large part of North America, and overlaps the current invaded range of European Gypsy Moth (*L*. *d*. *dispar*) (https://www.aphis.usda.gov/aphis/ourfocus/planthealth/plant-pest-and-disease-programs/pests-and-diseases/gypsy-moth). There are a number of countries in Africa and South America, which also have suitable climate. A number of these are either landlocked (e.g. Uganda, Zambia, Bolivia), do not have suitable climate on their coast (e.g. Kenya, Peru, Ecuador) or suitable climate at ports that receive international arriving ships (e.g. Angola). These countries would not be threatened by ships carrying AGM eggs. Further, there is little threat to south-eastern Asia, as the only locations in which climate appears suitable are found in non-coastal regions (Figure S1). Sensitivity and uncertainty analyses were conducted on the model parameters. The sensitivity analysis revealed that the potential establishment range was most sensitive to the Degree-days per Generation (PDD) parameter (Table S2). This parameter was informed by experimental data^16^ and the resulting modelled range limit accords well with distribution data. The uncertainty analysis (Figure S2) demonstrated a quite stable model. The areas of greatest geographical uncertainty extend into dry regions, and then only with low probability. The dry tolerance functions (Dry Stress parameters) are anchored in plant ecophysiology, where the concept of the permanent wilting point has been very well studied.

### Global shipping data

For all other countries, we identified the ports that fell within the climatically suitable range for AGM (vulnerable ports) and then calculated the proportion of ships that arrived into these vulnerable ports (Table 1). The countries threatened by AGM invasion via international shipping can be found in Africa, North and South America, as well Oceania (Table 1). We found the threat from these international ship arrivals varied from 0.01 (Costa Rica) up to 1.000 (Uruguay & New Zealand). Other countries with a high proportion of arriving ships presenting a potential risk include Canada (0.95) and Argentina (0.90). The total number of ships arriving to a vulnerable port should also be considered when assessing overall risk. Each ship has the potential to carry AGM eggs, so the more ships that arrive, the greater the threat. While the USA is ranked 7^th^ by proportion of ships that arrive to vulnerable ports, it receives more than 18,000 ships to those vulnerable ports, which is more than double the next ranked country (Canada). Other countries with a many ships arriving to vulnerable ports include those found in South America (Brazil, Argentina, and Venezuela) and Australia (all with more than 3,000 ships arriving to vulnerable ports).

**Table 1:** The proportion of international ship arrivals whose first port of call in the country is to a vulnerable port (i.e. a port where AGM is able to establish).

Country	Total ships	Ships to vulnerable ports	Proportion
Uruguay	1,233	1,233	1.00
New Zealand	2,031	2,031	1.00
Canada	8,069	7,662	0.95
Argentina	3,941	3,555	0.90
Chile	3,206	1,991	0.62
Venezuela	5,308	3,032	0.57
USA	35,432	18,415	0.52
Brazil	9,143	4,016	0.44
Australia	9,220	3,963	0.43
South Africa	5,533	1,735	0.31
Colombia	6,221	877	0.14
Cameroon	1,010	97	0.10
Mexico	7,140	424	0.06
Costa Rica	2,270	12	0.01

## Discussion

**Figure 2:**
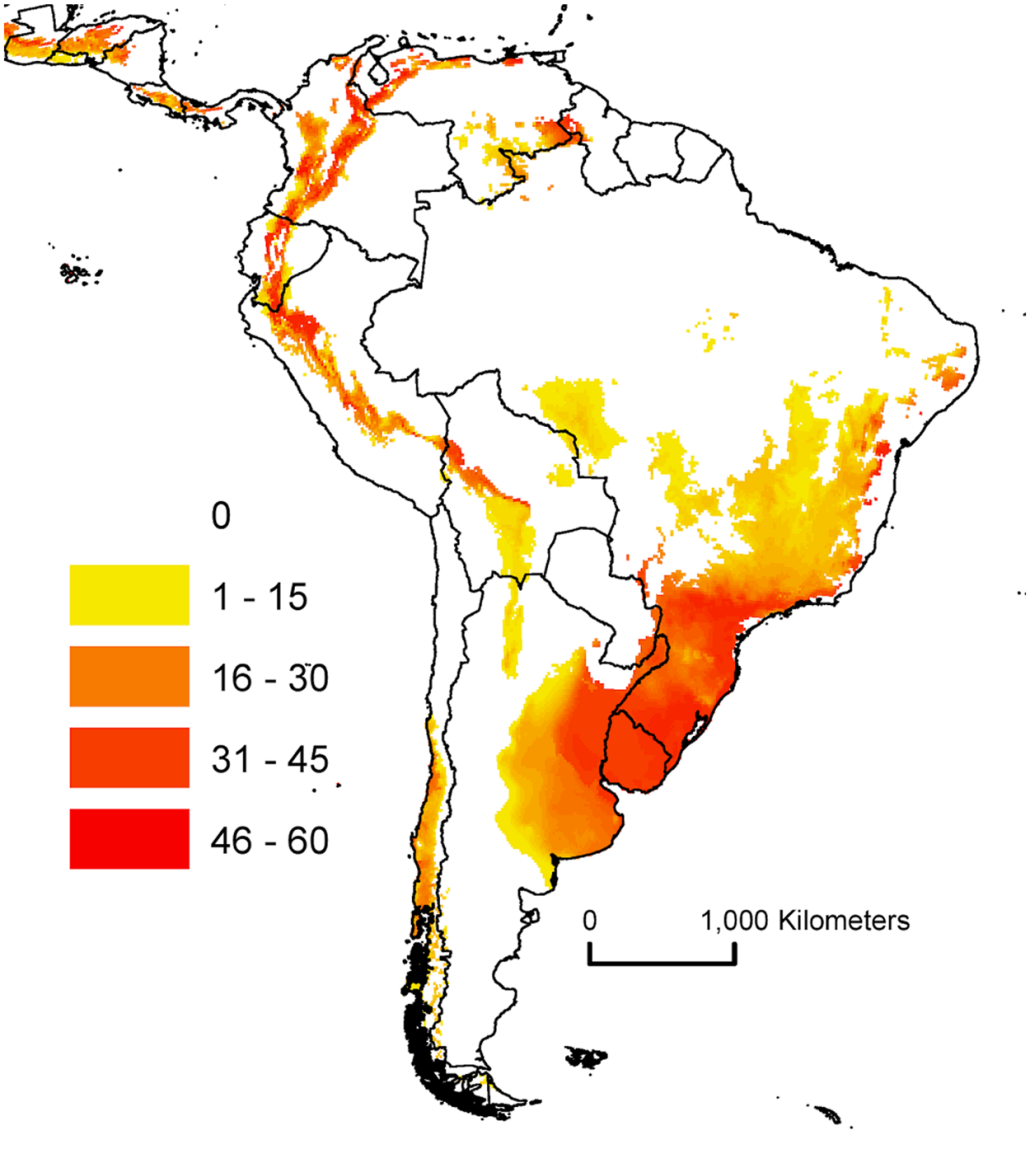
Climate suitability for gypsy moth (*L*. *d*. *asiatica* & *L*. *d*. *japonica*) in South America modelled using CLIMEX Ecoclimatic Index (EI).

**Figure 3:**
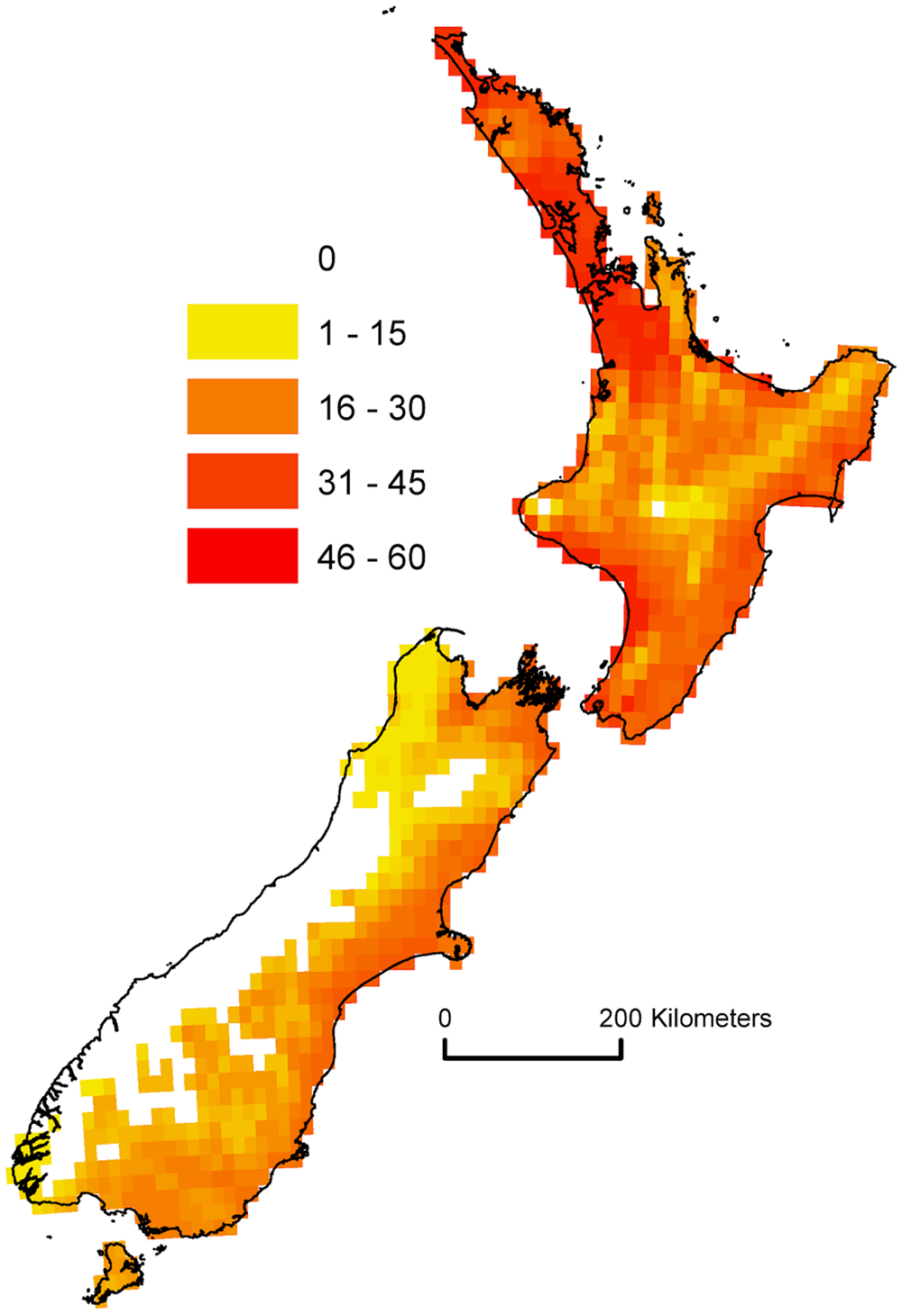
Climate suitability for gypsy moth (*L*. *d*. *asiatica* & *L*. *d*. *japonica*) in New Zealand modelled using CLIMEX Ecoclimatic Index (EI). Note all internationally arriving ships will arrive at a port found within the suitable modelled habitat.

At the global level, there is a large range in the proportion of ship arrivals that countries would need to consider as potential sources of AGM eggs. As can be seen from Fig. 1, for many countries, only specific regional coastal locations are suitable for AGM establishment. For example, southern Brazil, northern Argentina, and southern Australia all have ports that experience suitable climatic conditions for AGM establishment. Only international ship arrivals in those regions present a risk. For countries such as Uruguay (Fig. 2) and New Zealand (Fig. 3), *all* international ship arrivals present a potential threat, as all ports experience suitable climatic conditions for AGM. If a ship arrives into any port of these two countries carrying AGM eggs, and the larvae emerge upon arrival, the climatic conditions would likely be favourable for the establishment of a population.

Despite the apparent threats to these countries, not all arriving ships will present an equal threat level, as our analysis does not consider the likelihood of a ship carrying AGM eggs. Further refinement of any risk assessment could be achieved by identifying those ships that have previously berthed in ports located within the AGM native range (China, Japan, North and South Korea, and Far East Russia) during periods in which females are ovipositing (May to September^19,20^). Further, the number of nights a ship is berthed in a source port could also be considered, as this is the period when females oviposit. Finally, eggs need to complete diapause before they can hatch^21^. Gypsy moth diapause is complex, but ultimately dependent on temperature^21^. The temperatures egg masses are exposed to during an ocean voyage will determine the likelihood of having completed diapause upon arrival to a vulnerable port. A reliable method for estimating the likelihood of these eggs having completed diapause when they arrive to a vulnerable port, would considerably refine the estimated risk from incoming ships^12,14,15^. Initial analysis of ships arriving into Australia indicate that only a small proportion of those ships arriving into climatically suitable ports, will have spent some time at source ports during the AGM oviposition season, and if carrying eggs, those eggs would have actually completed diapause (unpublished data). Unfortunately, this level of shipping data detail at the global scale, was not available (see Methods) and so it was not possible to refine our analysis. However, this level of detail in shipping data reflects the level of detail available to many countries for internationally arriving ships (with the exception of USA, Canada, New Zealand, and more recently, Australia). For many of countries then, this analysis represents a ‘first step’ in identifying those ships of greatest threat of introducing AGM.

While the climate suitability maps presented here indicate where AGM populations could establish, a further refinement could consider the time of the year in which a ship arrives to a port and the weekly growth index (GI_W_) calculated in CLIMEX. This index (scaled from 0 to 1) estimates, on a weekly basis, if the conditions favour the growth of a population^22^. For example, Charleston (South Carolina), on the east coast of the USA, is a port within the potential distribution of AGM (Fig. 1). However, during the month of July, GI_W_ is zero due to excessive temperatures. Ships arriving during this period would likely present little risk as any larvae emerging from eggs on these ships are unlikely to survive. As discussed above, our shipping data did not include arrival dates (see Methods), and so it was not possible to refine the analysis in this way.

Interestingly, we find the maps generated from this analysis are considerably different to those from Peterson and colleagues^3^ (Figure S3). In particular, we note large tropical regions in Africa, South America and SE Asia that are identified as potentially suitable. While the authors state that temperate regions are likely suitable for establishment (presumably because only these regions showed ‘complete’ agreement between the 10 GARP models created), the published map shows a much larger area of potential distribution, which could be interpreted as such by readers. These differences have implications for some countries concerned with AGM populations establishing from incoming ships. For example, Peterson and colleagues show all of Brazil was identified as suitable (Figure S3), while our model reduced the suitable area to southern Brazil and subsequently, less than half of the arriving international ships (Fig. 2).

Our model also shows some differences with the Matsuki CLIMEX model^16^. In particular, for Australia, the EI map extends further up the eastern coast (Figure S4) than our CLIMEX model (Figure S5). Brisbane was also identified as being suitable, while our model does not. This has implications for the inspection of ships arriving into Brisbane from overseas. Brisbane received 1,087 international ship arrivals in 2005, which is approximately 10% of the total number of ships arriving into Australia, and the resources used to inspect ships arriving to Brisbane may not be necessary, or could be redirected to inspecting ships arriving to other Australian ports that fall within more suitable climates for AGM establishment (Figure S5). This conclusion is robust in the face of expected climate change, with climate suitability patterns shifting poleward in the face of a warming climate (data not shown).

This is the first global assessment of invasion threat via the shipping industry in the potential spread of gypsy moth (*L*. *d*. *asiatica* and *L*. *d*. *japonica*) and the range in threat can vary greatly from country to country. While national biosecurity agencies could use the outputs generated from this analysis, this is clearly just a first step to refining their inspection strategies and the deployment of resources. As a next step, we would recommend ship’s captains be required to provide a list of ports visited and dates prior to arrival (as is currently required for ships arriving to USA, Canada, New Zealand, Chile, and Australia). This information could be used to firstly, determine if a ship was present in a source port during the AGM oviposition period, and then, if eggs would have completed diapause upon arrival. Such information could greatly improve risk assessments by government agencies and limit the threat of invasion from this species via the global shipping network, while optimising inspection regimes and resourcing.

## Methods

### CLIMEX model

We used the computer-based niche modelling software program CLIMEX version 4^22^, which allows a semi-mechanistic niche model to be fitted inferentially based on a species current known distribution, and deductively using experimentally-derived species responses to climatic variables. These responses can also be inferred from phenological observations^23^. One of the main outputs generated by the CLIMEX model is an annual index of overall climatic suitability, the Ecoclimatic Index (EI). This value, scaled between 0 and 100, represents a combination of population growth during favourable conditions and survival during stressful conditions, where 0 represents unsuitable conditions and 100 represents optimal conditions for survival. The resulting map generated from EI represents the potential geographical distribution of a species as a function of climate. The detailed method used to generate a model can be obtained^22^, but briefly, the modeller sets the growth parameters based on available experimentally-derived knowledge, and then iteratively adjusts the stress parameters in the model until the EI map best matches the reported distribution for that species. Ideally, the model can be fitted to the known distribution in one continent and then validated against independent distribution data on other continents. Unfortunately, this was not possible for AGM as it is only found on one continent. Nonetheless, data or knowledge gained from different domains count as independent, and provides a means of cross-validation.

We combined distribution points from GBIF^24^ and the same distribution points used by Peterson and colleagues^3^ (Richard Williams pers comm.). Note, we removed from the Peterson *et al*.^3^ dataset one misleading point location, which was located in the Philippine Sea, approximately 900 km south of Japan, near the Island of Chichijima.

We used the base climatology data from CliMond 10’ with normals centred on 1975 (CM10_1975H_V1_1)^25^ and re-fitted the CLIMEX model using the parameters given in^16^. Temperature, moisture, cold stress, and heat stress parameters remained unchanged from Matzuki *et al*. (2001). We introduced wet stress and modified the diapause parameters. Wet stress limits the AGM distribution in southern Japan and is calculated as the difference between soil moisture (SM) and the wet stress threshold (SMWS) multiplied by the wet stress rate (HWS). The threshold for wet stress (SMWS) was set to 1.5, while the wet stress accumulation rate (HWS) was set to 0.05 week^−1^ (Table S1).

The diapause parameters used by Matsuki and colleagues^16^ generated an EI map, which did not match the reported AGM distribution. There was one eastern point in China (30.05 N, 102.0333E), which was not encompassed by the resulting model. In addition, the Matzuki model gave a potential distribution in China that extended further south than the reported distribution. We adjusted the average weekly minimum temperature that terminates diapause (DPT1) down from 7 °C to 5 °C in order to encompass the one eastern point in China. Increasing the minimum number of days to complete diapause (DPD) from 80 to 125 markedly improved the model concordance with the southern distribution limits of AGM in Asia (Table S1).

It should be noted that for another Gypsy moth subspecies (European Gypsy Moth – *Lymantria dispar dispar*) there has been a significant amount of research into the climatic factors that influence all stages of diapause (pre-diapause, diapause, and post-diapause) and there is a complex, non-linear relationship between temperature and progress through to diapause completion^21,26,27^. The detailed model of Gray, may operate similarly for AGM. However, CLIMEX operates at a broader spatial and temporal scale than Grays’ model, which broadly conforms to the CLIMEX winter diapause model insofar as it has a minimum temperature and presumably daylength that triggers the onset, a minimum duration, and warming conditions that break diapause. Hence, while the detailed behaviour of Gray’s model would be expected to differ, the broad pattern of behaviour across geographical scales would be expected to be similar.

We tested both sensitivity of the CLIMEX model parameters as well as the uncertainty in the EI values generated for the maps presented here. Sensitivity of the model parameters was evaluated by varying each parameter separately by the default values in CLIMEX^22^ (Table S2). The CLIMEX model is run where each parameter is adjusted up or down from the default value, and the effect on each state variable is recorded as the mean sum square change across all cells, or in the case of the range (EI > 0), the percentage of cells changing from suitable to unsuitable, or the reverse. These values are used to identify the most sensitive parameters.

Model uncertainty was tested by simultaneously selecting random values for each parameter from a triangular distribution using a Latin hypercube sampling scheme^22^. The range for each parameter is the same as indicated in the sensitivity analysis (Table S2). This was repeated for 100 simulations and a map was generated in which, for each cell, the number of simulations in which an EI > 0 was generated.

### Global shipping data

Global shipping data for 2005 were obtained from Keller and colleagues^28^. These authors purchased this data from Lloyds Maritime Intelligence Unit, which manages a global data set collected by the Lloyd’s of London Agency Network. While this database contains an extensive list of port locations and ship movement data at the global level, it is licensed and therefore protected from general distribution. As such, Keller and colleagues were not able to provide extensive detail of the data they analysed (e.g. ship IMO, dates of port arrival and departure). However, they were able to provide the number of voyages travelling between any two ports at the global level, which we used for our analysis. For any country’s port, we only considered those ships arriving from an international port. Those ships arriving from a domestic port, are either domestic-only ships, or they would have previously arrived to an another port within that country from a foreign port (we assumed that an assessment of a ship arriving from an international port would be assessed for its risk of carrying AGM eggs at the first port of call to that country). Because date of arrival data were not made available, it was not possible to filter the number of ships arriving to the climatically suitable period in which AGM would be able to establish.

In addition, we considered all ships arriving from any international port as potentially carrying AGM eggs. While a many of these ports will not be the location in which a ship received AGM eggs (because AGM populations are not found there), it is possible that the ship previously visited a port where AGM populations are found. As mentioned above, the data we used did not give ship details and so it was not possible to identify the pathways a ship travelled prior to the last international port before arriving into a country’s port. As such, we assumed any arriving ship had the potential to have visited a source port and be carrying AGM eggs. This also often reflects the level of detail a country’s port authority will have for any internationally arriving ship (with the exception of USA, Canada, Australia, and New Zealand, who specifically ask for more detailed port visitation data for internationally arriving ships).

AGM eggs can be transported on ships, shipping containers or vehicles^19^. Evaluating the risk from shipping containers and imported vehicles is difficult, particularly at the global level, where it is not possible to obtain data on where containers and vehicles are transported to after being unloaded from a ship. As such, we restricted our assessment to the risk only from ships that may be carrying eggs. Any larvae that might hatch from these eggs could only disperse a few kilometres through ballooning^29^. We therefore identified those ports found within climatically suitable coastal areas, as defined by a CLIMEX model. The proportion of ships to these ports from international sources were then used to identify those countries with the greatest threat from AGM establishment. Finally, we did not consider risk of invasion to countries found within the native range of European Gypsy moth (i.e. Europe and North Africa).

The datasets generated during and/or analysed during the current study are available from the corresponding author on reasonable request.

## Electronic supplementary material

Supplementary information
