# Supplementary material for: Global establishment threat from a major forest pest via international shipping: Lymantria dispar
Source: Sci Rep. 2018 Sep 13;8:13723. doi: 10.1038/s41598-018-31871-y (PMC6137135; doi:10.1038/s41598-018-31871-y)
Supplement: Supplementary file 1 — Supplementary information [file 41598_2018_31871_MOESM1_ESM.docx]

**Global establishment threat from a major forest pest via international shipping: *Lymantria dispar***

Paini, D.R., Mwebaze, P., Kuhnert, P.M., Kriticos, D.J.

**Supplementary Information**

Table S1. Changes in the CLIMEX *L. dispar* model developed by Matzuki ^1^. Those parameters that have been altered are in bold. Refer to methods for explanation of changes and additions.

| CLIMEX parameters | Matzuki et al (2007) | Present values |
| --- | --- | --- |
| Temperature |  |  |
| DV0 | 1 | 1 |
| DV1 | 14.5 | 14.5 |
| DV2 | 28 | 28 |
| DV3 | 32 | 32 |
| PDD | 1700 | 1700 |
| Moisture | | |
| SM0 | 0.2 | 0.2 |
| SM1 | 0.3 | 0.3 |
| SM2 | 1.3 | 1.3 |
| SM3 | 1.5 | 1.5 |
| Diapause | | |
| DPD0 | 14.5 | 14.5 |
| DPT0 | 19 | 19 |
| **DPT1** | **7** | **5** |
| **DPD** | **80** | **125** |
| Cold Stress | | |
| TTCS | -20.0 | -20.0 |
| THCS | -0.0005 | -0.0005 |
| Heat Stress | | |
| TTHS | 32 | 32 |
| THHS | 0.005 | 0.005 |
| Wet Stress | | |
| **SMWS** | **Not used** | **1.7** |
| **HWS** | **Not used** | **0.05** |


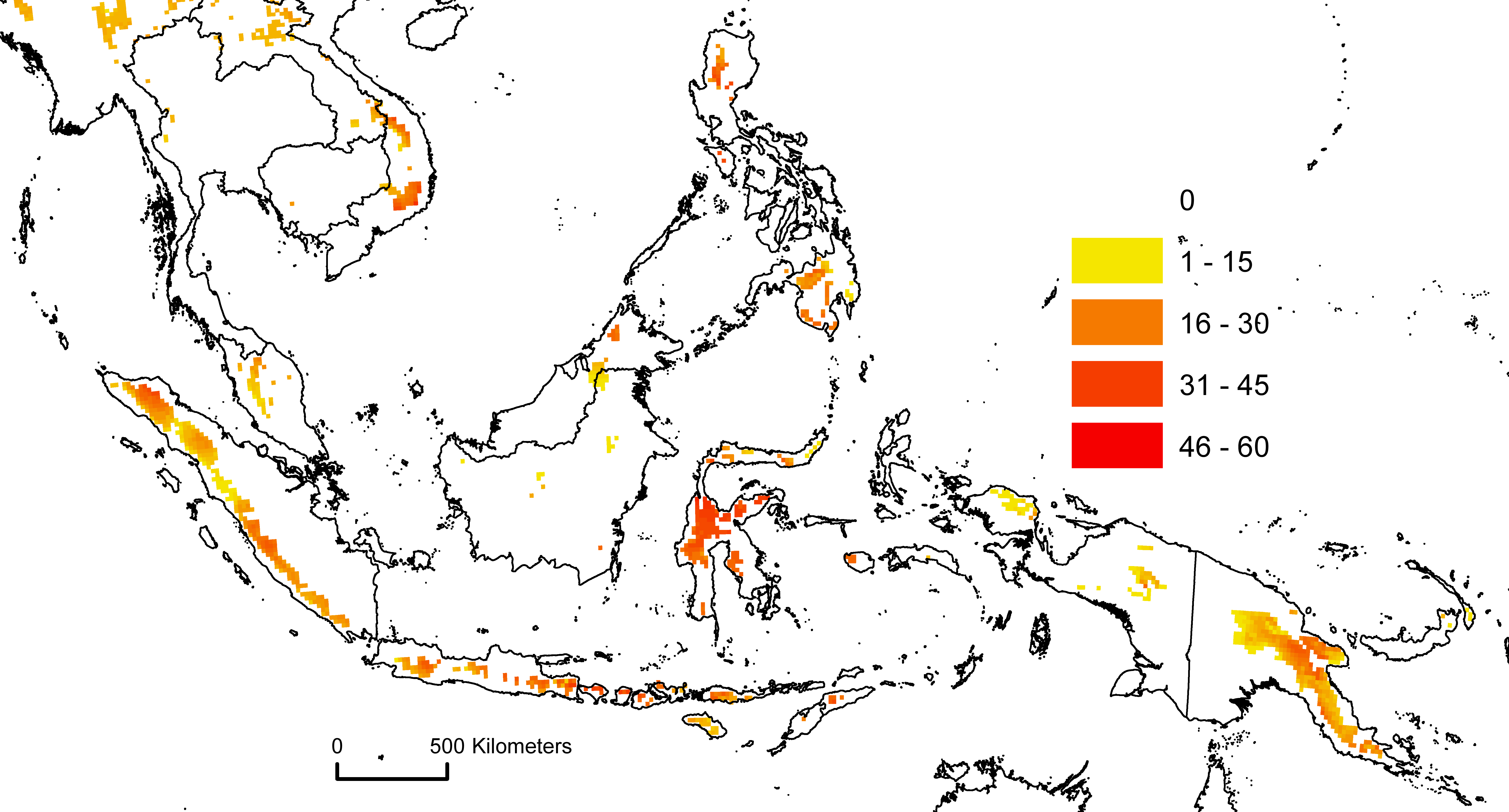


Figure S1. Climate suitability for gypsy moth (*L. d. asiatica* & *L. d. japonica*) in South East Asia modelled using CLIMEX Ecoclimatic Index (EI).


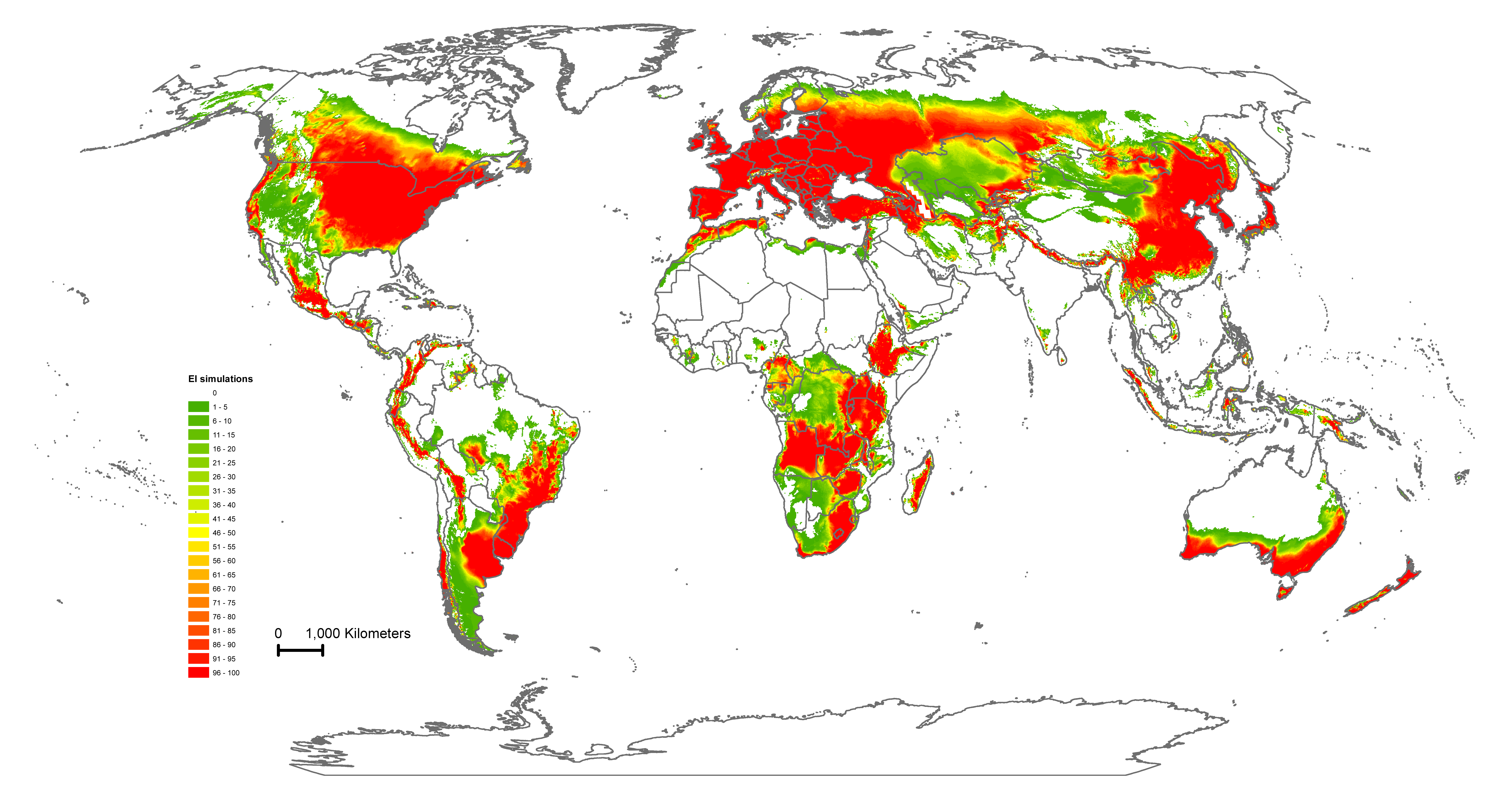


Figure S2. Results of the uncertainty analysis. 100 simulations were run and for each cell and the percentage of simulations in which EI>0 was calculated. Compare with Figure 1.


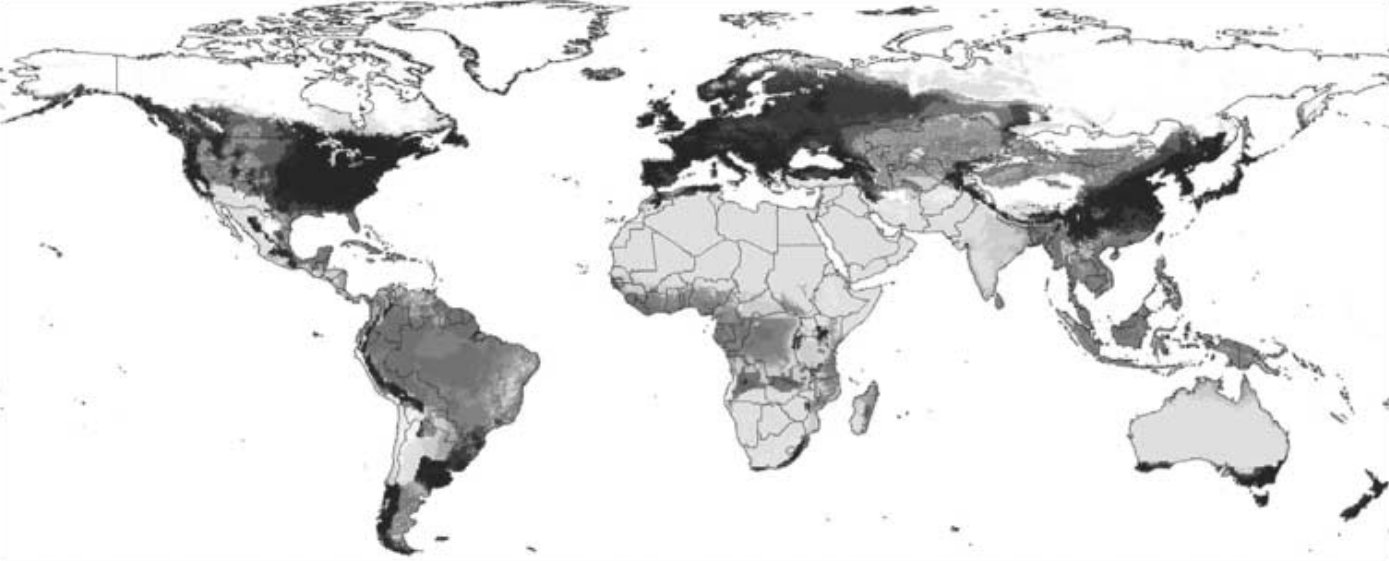


Figure S3. Global map of modelled AGM potential distribution from (Peterson et al. 2007) (reproduced with permission)

Figure S4. CLIMEX model for AGM potential distribution in Australia (Matsuki et al. 2001) (refer Figure S5 for comparison) (reproduced with permission)


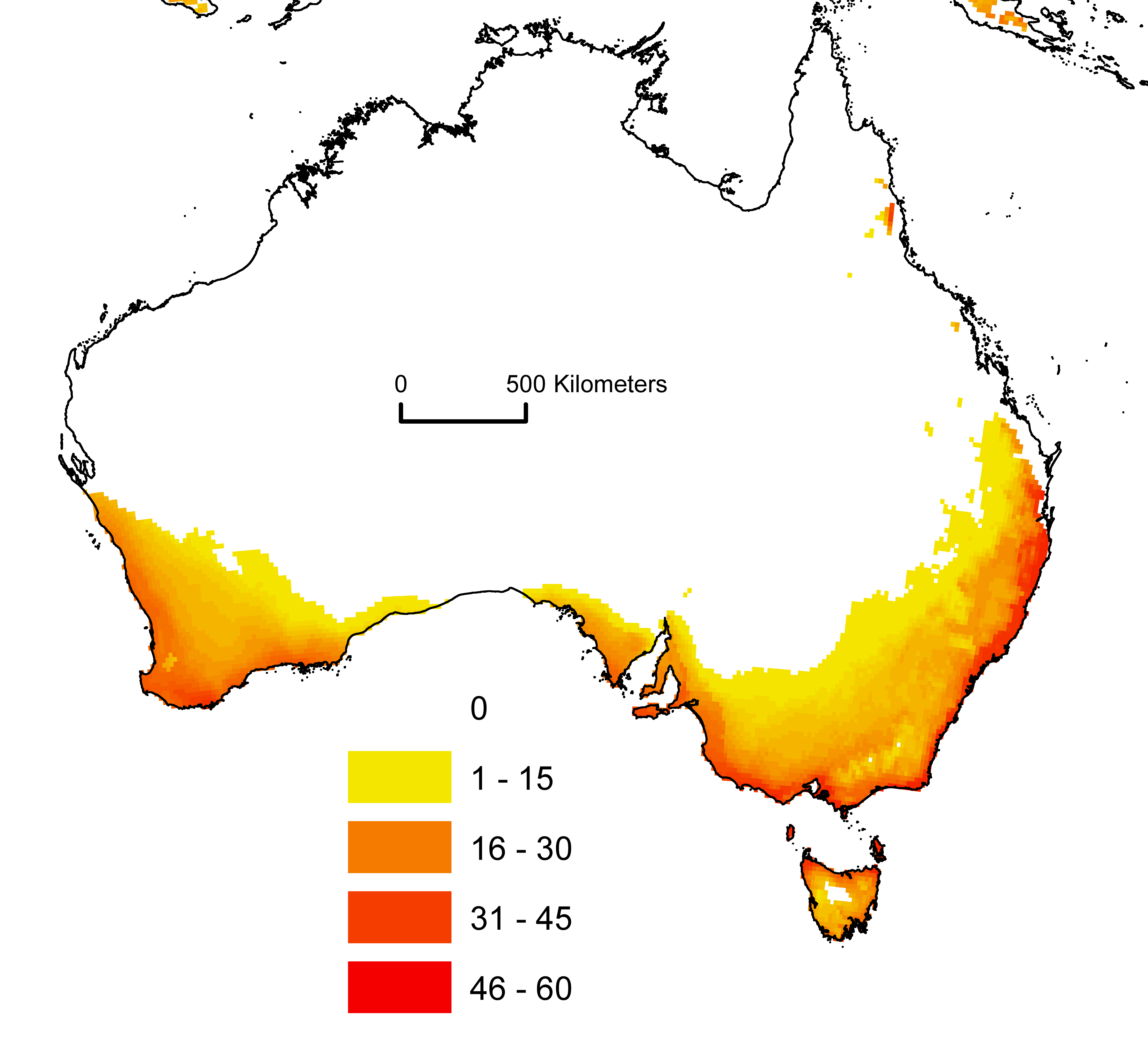


Figure S5. Climate suitability for gypsy moth (*L. d. asiatica* & *L. d. japonica*) in Australia modelled using CLIMEX Ecoclimatic Index (EI), showing no potential peri-coastal establishment north of Brisbane (refer Figure S4 for comparison)

Table S2. Results of the sensitivity analysis on parameters in the CLIMEX model. Range change is the percentage of cells that were altered from suitable to unsuitable (or the reverse). EI change is the mean sum square change across all cells.

**References**

Peterson, A. T., Williams, R. & Chen, G. Modeled global invasive potential of Asian gypsy moths, Lymantria dispar. *Entomologia Experimentalis et Applicata* **125**, 39-44 (2007).

Matsuki, M., Kay, M., Serin, J., Floyd, R. & Scott, J. K. Potential risk of accidental introduction of Asian gypsy moth (Lymantria dispar) to Australasia: effects of climatic conditions and suitability of native plants. *Agricultural and Forest Entomology* **3**, 305-320 (2001).
